# Prophylactic treatment uptake and compliance with recommended follow up among HIV exposed infants: a retrospective study in Addis Ababa, Ethiopia

**DOI:** 10.1186/1756-0500-4-563

**Published:** 2011-12-27

**Authors:** Mulatu Biru Shargie, Frida Eek, Addisalem Abaychew

**Affiliations:** 1 Master's Programme in Public Health Faculty of Medicine Lund University Malmö, Sweden; 2 Division of Laboratory medicine/Occupational and Environmental Medicine, Lund University, Lund, Sweden; 3 Department of Nursing, Bethel Medical College, Addis Ababa, Ethiopia

## Abstract

**Background:**

Children are being infected by HIV/AIDS mainly through mother-to-child transmission. In Ethiopia currently more than 135,000 children are living with HIV/AIDS. The aim of this study was to describe the pattern of ARV uptake after birth, co-trimoxazole prophylaxis and follow up compliance, and to examine which factors are associated with the intervention outcome.

**Methods:**

A retrospective quantitative study design was used for data collection through two hospitals. All infants who were delivered by HIV infected mothers between October 2008 and August 2009 were included and information regarding treatment adherence during their first 6 months of age was collected.

**Findings:**

118 HIV exposed infant-mother pairs were included in the study. 107 (90.7%) infants received ARV prophylaxis at birth. Sixty six (56%) of the infants were found to be adherent to co-trimoxazole prophylactic treatment. The majority (*n *= 110(93.2%)) of infants were tested HIV negative with DNA/PCR HIV test at the age of sixth weeks. Infants who took ARV prophylaxis at birth were found to be more likely to adhere with co-trimoxazole treatment: [OR = 9.43(95% CI: 1.22, 72.9)]. Similarly, infants whose mothers had been enrolled for HIV/ART care in the same facility [OR = 14(95% CI: 2.6, 75.4)], and children whose fathers were tested and known to be HIV positive [OR = 3.0(95% CI: 1.0, 9.0)] were more likely to adhere than their counterparts. Infants feeding practice was also significantly associated with adherence *χ*^2 ^-test, *p *< 0.01.

**Conclusion:**

The proportion of ARV uptake at birth among HIV exposed infants were found to be high compared to other similar settings. Mother-infant pair enrolment in the same facility and the infant's father being tested and knew their HIV result were major predictors of infants adhering to treatment and follow up. However, large numbers of infants were lost to follow up.

## Background

Children under 15 years of age are being infected by human immunodeficiency virus (HIV) mainly through mother-to-child transmission (MTCT). According to World Health Organization (WHO) 2007 report daily HIV infection among children less than 15 years old is estimated to be 1,500 [1]. In low income countries, especially in Africa, all children born by HIV infected mothers are supposed to receive co-trimoxazole (trimethoprim-sulfamethoxazole) prophylaxis to prevent the occurrence of *Pneumocystis jiroveci *pneumonia (PCP) from 6 weeks of age and onwards, until the child gets tested and determined HIV negative [2]. This prophylaxis is found to be very effective in decreasing high number of death due to PCP among infants and children with HIV, especially in poor countries where direct viral assessment is expensive and instead antibody-based HIV test is used. Moreover; in low income countries it is still difficult to determine the HIV test result among new born neonates and infants with direct viral assay test, due to the fact that the majority of infants have no good alternative to breast milk, which increase the risk for HIV due to breast feeding from HIV infected mothers. Therefore, as long as a child is breast fed, co-trimoxazole is indicated until the infant or child is no longer at risk to acquire HIV from breast milk [3,4].

Ethiopia is one of the low-income countries in sub-Saharan Africa that suffer hard from the HIV epidemic. In Ethiopia, it was estimated that there were 135,000 HIV infected children in 2005 [5]. Despite great number of children born by HIV infected mothers, only 2% of HIV infected mothers were found to pass through prevention of mother-to-child HIV transmission (PMTCT) intervention in 2005. PMTCT guidelines were developed in Ethiopia only recently, in 2001. However, due to lack of free access of anti retroviral (ARV) prophylaxis in the country until 2005, the challenge remained even after the guideline has developed. In 2005, free ARV was launched to everybody who was in need [6]. Currently, the country is implementing the PMTCT program with the minimum package, which includes the regular provision of HIV counselling and testing, safe and quality obstetrical services, provision of HIV care for mothers, ARV prophylaxis for mother and infant when indicated, counselling on infant feeding options, family planning and strengthened referral linkage.

The provision of PMTCT prophylaxis to HIV positive women is allocated according to set criteria. If a pregnant mother is eligible to start antiretroviral therapy (ART), she is supposed to start the long-term treatment of a combination of triple highly active antiretroviral therapy (HAART) after the end of first trimester, which has a great role in preventing mother-to-child HIV transmission. For pregnant women who are not eligible for ART provision, prophylaxis should be started with zidovudine (AZT 300 mg 2×/day), starting at 28 weeks of pregnancy or as soon as possible thereafter. During onset of labour and delivery, triple ARV should be given and a combination of two ARV should continue for 7 days. The HIV exposed infant should also be given a single dose of neverapine (NVP) at birth and AZT for 7 days. "The AZT dose for the infant should be extended for 4 weeks if a mother didn't receive adequate dose less than 4 weeks before delivery" [6]. Despite several activities being implemented in Ethiopia, both ARV uptake and monthly follow up compliance among infants is currently not satisfactory. The majority of infants are lost to follow-up before the final HIV infection status is determined [7].

### The aim of the study

To describe the pattern of ARV prophylactic treatment uptake after birth as well as the follow up compliance of taking co-trimoxazole prophylaxis, and to examine which factors are associated with intervention outcome.

## Methods

### Research design and methods

The study was conducted using a retrospective quantitative study design with retrospective data collection, using the registration books and follow up logs in the PMTCT and HIV exposed infants' follow-up units.

### Study setting and participants

The study was carried out among infants enrolled to HIV exposed infants follow up program in Zewuditu Memorial and Yekatit 12 Hospitals, in Addis Ababa, the capital of Ethiopia. The total population of Addis Ababa is 3 million, accounting for 3.7% of the country's population [8]. Zewuditu memorial and Yekatit 12 hospitals serve patients with referral slips from all over the country. However, HIV exposed infants mainly come from Addis Ababa or, to some extent, from the surrounding districts. The source populations for this particular study were infants who had been enrolled to HIV exposed infants follow up in the facility. The study population included the cohort of HIV exposed infants who were delivered between October 2008 and August 2009 in the two hospitals, and not suffering from any other known severe illness. 131 HIV exposed infants were delivered during the given period of time. Thirteen infants were found to lack a complete record about required variables, and were therefore excluded. The final study sample consisted of 118 HIV exposed infants.

### Data collection approach and instrument

The data collection was conducted from February 15 to March 5, 2010 within the specialized referral hospitals of Zewuditu memorial and Yekatit 12 Hospitals in Addis Ababa, Ethiopia. Information regarding treatment adherence during their first 6 months of age was collected from registration books (see below). At the last follow up visit, the sixth month of infants' treatment adherence and the first 6 months follow up compliance were observed from the follow up registration books during the data collection period at both referral hospitals. The main source of information for this particular study was the registration book for the HIV exposed infants follow-up. PMTCT pregnant mothers' registration log books were also investigated regarding the infant mother's socio-demographic data (age, current marital status, number of children she have, occupation, educational status, her partner live and HIV status) as well as her enrolment for care and support within the hospital.

Some of the infants mothers were interviewed via telephone by hospital nurses to complete some missing socio-demographic variables include age, current marital status, educational status, occupational status and the number of children she has. A structured template was prepared to collect the relevant information from the registration books. Two clinical nurses in each specialized referral hospital supported the principal investigator in the process of collecting relevant information about the HIV exposed infant-mother pair from the registration books.

### Data analysis and processing

All data analyses were performed using Statistical Package for Social Science (SPSS window version 15.0). Frequency distribution of socio-demographic and economic variables of HIV exposed infants and their mothers' pair and some socio-demographic variables of infant's father are presented. Cross tabulation and chi-square test was performed in order to compare and determine the differences in predictors among treatment adherent and non-adherent HIV exposed infants. In a first step, we used chi square/fisher's test to look for difference in the distribution between groups. If chi square indicated significant difference in distribution between the two groups, a logistic regression were performed to further explore the relationship between co-trimoxazole treatment adherence and socio-demographic factors. Unadjusted as well as adjusted models were tested. The adjusted models included potential confounders such as mothers' age, education, occupation, mother's enrollment for HIV/ART care and the place where mother's enrolled for HIV/ART care. Statistical tests was determined and interpreted for statistical significance with *p*-values < 0.05 considered significant. Odds ratios are presented with corresponding 95% confidence intervals. The summarized results are presented using tables and graphs.

### Operational definitions

#### Adherence for co-trimoxazole prophylaxis

A child is said to be adherent if he/she missed no more than three doses (took more than 95% of the prescribed doses correctly) for 1 month prior to the study. Children who were lost to follow up were also considered as non-adherent to co-trimoxazole treatment.

#### Follow-up compliance

HIV exposed infants who were enrolled to follow up unit at any time of the first 6 months of their age in the given time duration and followed the remaining visits regularly were defined as compliant.

#### Infants' antiretroviral prophylactic treatment uptake

An infant having single dose NVP at birth and AZT for 7 days.

#### HIV-concordant couples

Both mother and father of the child having the same HIV test result, either HIV positive or negative. (In this particular study it means that both couples should be HIV positive to fulfil the definition, since all included mothers were HIV positive).

#### HIV-discordant couples

Mother and father of the child having different HIV test results (in this case meaning that the infant's mother is HIV positive and father is HIV negative)

#### Ethical Considerations

Ethical approval was secured from Addis Ababa City Administration Health Bureau and Zewuditu memorial and Yekatit 12 specialized hospitals. The study was strictly followed "the international principles of research ethics outlined in the world medical association's Declaration of Helsinki (2004)".

## Findings

### Socio-demographic and economic characteristics

**Figure 1:**
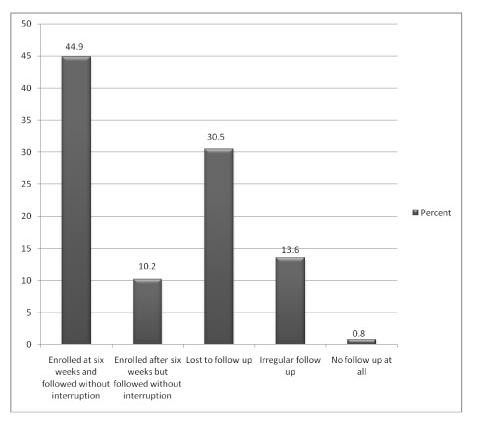
**HIV Exposed Infants Age of Enrollment versus Follow up Compliance in Addis Ababa, Ethiopia March 2010**.

Socio demographic characteristics of the 118 HIV exposed infants and their parents who were included in the study presented in Table 1. Figure 1

**Table 1:** Socio-Demographic characteristics of HIV exposed infants, their mothers and fathers in Addis Ababa, Ethiopia (n = 118), March 2010

Variables	Frequency(percentage)
Sex of the Infant	

Boy	65(55.1)

Girl	53(44.9)

Infants age of enrollment	

At 6 weeks	99(83.9)

After 6 weeks	19(16.1)

Infants Received ARV prophylaxis at birth	

Yes	107(90.7)

No	11(9.3)

Infants feeding practice	

Exclusive Breast feeding	52(44.1)

Replacement feeding	61(51.7)

Mixed feeding	5(4.2)

Infants PCR/DNA HIV test result	

Negative	110(93.2)

Positive	5(4.2)

Unknown	3(2.5)

Age of the mother (Mean = 28.15)	

< 25	22(18.6)

25-28	32(27.1)

28-31	34(28.8)

> = 31	30(25.4)

Marital status of the mother	

Married	73(61.9)

Unmarried	29(24.6)

Divorced	10(8.5)

Widowed	6(5.1)

Educational status of the mother	

Unable to read and write	8(6.8)

Primary education 1-8	50(42.4)

Secondary education 9-12	51(43.2)

Diploma and above	9(7.6)

Occupational status of mother	

Employed	11(9.3)

Self employed	28(23.7)

Unemployed	79(66.9)

Number of children a mother have	

1-2	37(31.4)

3-5	81(68.6)

Mother enrolled in HIV/ART care	

Yes	105(89)

No	13(11)

Where the mother enrolled for care	

In the facility	86(72.9)

Out of the facility	11(9.3)

Unknown	21(17.8)

Primary Care taker	

Biological	115(97.5)

Non-Biological	3(2.5)

Father HIV status	

Positive	62(52.5)

Negative	20(16.9)

Unknown	36(30.5)

Father live status	

Alive	113(95.8)

Dead	5(4.2)

### Distribution of potential predictors among adherent and non-adherent groups

Differences in distribution of potential predictors between adherent and non-adherent groups were explored using chi-square test. Some variables such as ARV prophylaxis taken by infants at birth, infants' mothers' place of enrolment for their HIV/ART care and infants with their fathers who were tested for HIV were found to be associated with adherence of recommended treatment for co-trimoxazole prophylaxis (Table 2).

**Table 2:** Differences in co-trimoxazole treatment adherence among HIV exposed infants through various categorical variables (*n *= 118), March, 2010

Variables	Adherent n(%)	Non-adherent n(%)	Chi-Square (*χ*^2^) tests value	Df	P-value
Infant Received ARV prophylaxis at birth					

Yes	63(95.5)	44(84.6)	4.04	1	^f^0.04

No	3(4.5)	8(15.4)			

Infant feeding practice					

Exclusive breast feeding	37(56.1)	15(28.8)	10.3	2	^L ^0.006

Replacement feeding	28(42.4)	33(63.5)			

Mixed feeding	1(1.5)	4(7.7)			

Current marital status of the mother					

Married	37(56.1)	36(69.2)	2.14	1	0.14

Unmarried	29(43.9)	16(30.8)			

Where the mother enrolled for care					

In the facility	56(84.8)	30(57.7)	14.54	2	0.001

Out of the facility	6(9.1)	5(9.6)			

Unknown	4(6.1)	17(32.7)			

Number of children the mother has					

1-2	25(37.9)	12(23.1)	2.96	1	0.08

3-5	41(62.1)	40(76.9)			

Father HIV status					

Positive	39(59)	23(44)	6.13	2	0.04

Negative	13(20)	7(14)			

Unknown	14(21)	22(42)			

Df = degree of freedom; ^f ^= fisher's exact test; ^L ^= Likelihood ratio

Other factors including infants' sex, age of enrollment, place where the infant was referred from, age of mother, educational & occupational status of mother, mothers HIV/ART care enrollment, number of children a mother have and mothers who provided PMTCT were not found to be associated with adherence.

### Factors associated with infants' treatment adherence

Some variables were found to be significantly associated with adherence of recommended treatment for co-trimoxazole prophylaxis after controlling for potential confounders (Table 3). Significantly increased OR's for adherence were found for children who received ARV prophylaxis at birth, whose mothers were enrolled for care in the facility, and who had HIV concordant parents, i.e. both mother and father were HIV positive.

**Table 3:** Independent predictors of adherence to co-trimoxazole prophylaxis among cohort of HIV exposed infants Addis Ababa, Ethiopia [*n *= 118], March, 2010

Variables	cOR^a ^(95% CI)	aOR^b ^(95% CI)
Infant Received ARV prophylaxis at birth		

Yes	3.8(1.0, 15.2)	* 9.4(1.2, 72.9)

No	1	1

Where the mother enrolled for care		

In the facility	7.9(2.5, 25.7)	*14(2.6, 75.4)

Out of the facility	5.10(1.02, 25.54)	7.1(0.9, 60)

Unknown	1	1

Father's HIV status		

Positive (concordant)	2.5(1.1, 6.2)	*3.0(1.0, 9.0)

Negative(discordant)	2.9(0.9, 9.1)	3.5(0.8, 14.6)

Unknown	1	1

cOR^a ^= Crude Odds Ratio, aOR^b ^= Adjusted Odds; CI = Confidence Interval; * P-value < 0.05

A greater proportion of infants were found to be adherent with treatment among children whose mothers had primary or secondary education; 42.4% and 41% respectively, compared to only 6% among children whose mother were unable to read and write. However, mothers' education was not found to be significantly associated with adherence.

## Discussion

Studies have shown that ARV treatment uptake at birth and follow up compliance with recommended treatment among infants and children, especially in resource limited countries, are faced with tremendous challenges [9-11]. In this study, HIV exposed infants ARV prophylaxis uptake at birth found to be 91%. This is high compared to similar studies conducted in Zimbabwe and Uganda, where ARV prophylaxis uptake was found to be 31% and 84.8% respectively [9,12]. Possible reasons for better ARV treatment uptake among HIV exposed infants in our study might be that Ethiopia has an integrated and comprehensive care and follow up program, as well as an improved service quality in Addis Ababa health facilities. A further reason could be an increased awareness about the importance of PMTCT intervention among infants' parents.

Even though a great proportion of infants had received ARV prophylaxis at birth, about 31% of them were eventually lost to follow up and their co-trimoxazole treatment had been interrupted. One of the reasons for this could be that the infants' mothers were being faced with various difficulties while following a specific care program laid out for the child, and perhaps a fear that treatment in the facility would lead to stigmatization and discrimination by the community or their partners. Another reason might be that when the infants' first PCR/DNA HIV test result (at 45 days of age) is negative, the mother may decide that it is not necessary to continue follow up further, or may change their address without informing the facility. A further reason could be lack of charge free co-trimoxazole drug supply in the stock of the facility, so treatment cannot be provided regularly without interruption. A similar study conducted in Uganda indicated that 53% of mother-infant pairs were lost-to-follow up (LTFU) [12]. The major possible reasons discussed in this study were lack of awareness about the importance of follow up among the mothers, risk of death and lack of male partner involvement through the ongoing care and treatment package being implemented in the facility [12,13].

Despite a large uncertainty in the estimation, our study indicated that enrollment of mothers in HIV/ART care and support in the same facility where HIV exposed infants attend their regular follow up was associated with 14 times higher odds for infants to adhere with treatment, compared to enrollment in "unknown" facility (95% CI, 2.6, 75.4). A very recent study conducted in Abidjan Cote d'Ivoire has shown that HIV exposed infants' mothers' enrollment in care through family-focused model of HIV care in the facility increased infants' treatment follow-up compliance. In addition, this program was found to be successful in promoting the involvement of male partners and other family members in HIV care, as the program was addressing the needs of all individuals in the family [14].

As indicated in our study results, infants whose fathers were tested for HIV with known result, mainly concordant with mothers', were found to be 3 times higher odds for infants to adhere with treatment than those who didn't know their HIV status (95% CI, 1.0, 9.0). A recent similar study conducted in Zimbabwe indicated that male partners' involvement in HIV testing is very low, which may affect the PMTCT and voluntary counseling and testing (VCT) service uptake among HIV positive pregnant mothers and, subsequently, their HIV exposed infants [9].

Regarding infants PCR/DNA HIV test outcome at their age of sixth week, 93% of HIV exposed infants in our study found to be HIV negative. Hence, among the cohort of HIV exposed infants only 4% were found to be HIV positive during their sixth week PCR/DNA test and the remaining 3% were with unknown HIV test outcome. The relatively low number of HIV infected infants in our study could be due to enhanced access to services among HIV infected mother-infant pair and the effectiveness of PMTCT intervention during pregnancy, intra-partum and post-natal period with all other comprehensive PMTCT intervention packages. In resource-rich settings, the risk of HIV transmission from HIV infected pregnant mother-to-infants has already declined to less than 2%, due to good access of comprehensive PMTCT package for infants [15-17]. Other studies conducted in various countries of Africa have shown that HIV test result disclosure and male-partners involvement on HIV/AIDS prevention, care and support package through community mobilization and behavioral change communication, promoted both the HIV exposed infant-mother pairs PMTCT intervention uptake as well as adherence with prophylactic treatment [18-20].

In this study, infants' treatment and follow-up adherence was assessed along with their parents' socio-demographic and health care utilization characteristics to determine the main predictors associated with treatment and follow up adherence, using chi-square test and unadjusted and adjusted logistic regression analyses. The reason for using adjusted analyses was to explore the effects of various predictors on the outcome, after controlling for potential confounding factors.

### Limitations and strengths

We have faced some limitations while conducting and analyzing this study. Several subjects were lacking a complete record of required variables in the data source, which hampered the size of our study sample and led to large statistical uncertainty. Another limitation with this study was that infants' adherence for treatment through both hospitals was determined using a record that was completed by health providers based on the information provided by infants' mothers, and by searching the follow up compliance of infants based on their appointment dates. The information gathered from infants' mothers may not be perfectly valid, and the prevalence of adherence might hence have been overestimated. If the subjects had been directly interviewed and if clinical markers had been applied, the information about infants' treatment adherence may perhaps have been more accurate.

Despite the limitations identified and explained, this study has several strengths. As there is no adequate study about HIV exposed infants follow up and prophylactic treatment compliance, especially in Ethiopia, it adds some valuable information. The findings of this study provide additional information about the process of HIV exposed infants' service provision and follow up. Therefore, this study contributes to the HIV exposed infants follow-up guidelines of the country, since no such investigations have been performed so far in the country despite it being an area with many challenges shared by other sub-Sahara Africa countries [11]. The study also provides a good opportunity to give feedback on the experience and trend of the HIV exposed infants' follow-up in the facility, which is important for further planning and evidence-based decision making.

## Conclusion

The proportion of ARV prophylaxis uptake among the cohort of HIV exposed infants in Addis Ababa included in this study was found to be high. However, a great number of infants were found to be lost to follow up. ARV prophylaxis at birth, place of mothers' enrollment for care, and fathers' HIV status were significantly associated with treatment adherence among children, although the precision in the estimated OR's were low. Finally we recommend a further large-scale study to explore the challenges related to the follow-up of HIV exposed infants.

## Competing interests

The authors declare that they have no competing interests.

## Authors' contributions

MB developed and designed the idea of study, performed the data analysis, interpretation and wrote the manuscript. FE assisted in all stages of this study include with the stage of design, critical data analysis, interpretation and final review of the manuscript. AA assisted during the design of the study, data analysis and interpretation All authors read and approved the final manuscript.
